# Automatic glioma segmentation based on adaptive superpixel

**DOI:** 10.1186/s12880-019-0369-6

**Published:** 2019-08-23

**Authors:** Yaping Wu, Zhe Zhao, Weiguo Wu, Yusong Lin, Meiyun Wang

**Affiliations:** 1 School of Electronic and Information Engineering, Xi’an Jiaotong University, Xi’an, 710049 Shaanxi China; 2 Collaborative Innovation Center for Internet Healthcare & School of Software and Applied Technology, Zhengzhou University, Zhengzhou, 450052 Henan China; 3 Department of Radiology, Henan Provincial People’s Hospital, Zhengzhou, 450003 Henan China

**Keywords:** Glioma segmentation, Superpixel, MRI, Medical image processing, Machine learning

## Abstract

**Background:**

The automatic glioma segmentation is of great significance for clinical practice. This study aims to propose an automatic method based on superpixel for glioma segmentation from the T2 weighted Magnetic Resonance Imaging.

**Methods:**

The proposed method mainly includes three steps. First, we propose an adaptive superpixel generation algorithm based on simple linear iterative clustering version with 0 parameter (ASLIC0). This algorithm can acquire a superpixel image with fewer superpixels and better fit the boundary of region of interest (ROI) by automatically selecting the optimal number of superpixels. Second, we compose a training set by calculating the statistical, texture, curvature and fractal features for each superpixel. Third, Support Vector Machine (SVM) is used to train classification model based on the features of the second step.

**Results:**

The experimental results on Multimodal Brain Tumor Image Segmentation Benchmark 2017 (BraTS2017) show that the proposed method has good segmentation performance. The average Dice, Hausdorff distance, sensitivity, and specificity for the segmented tumor against the ground truth are 0.8492, 3.4697 pixels, 81.47, and 99.64%, respectively. The proposed method shows good stability on high- and low-grade glioma samples. Comparative experimental results show that the proposed method has superior performance.

**Conclusions:**

This provides a close match to expert delineation across all grades of glioma, leading to a fast and reproducible method of glioma segmentation.

## Background

Glioma is a prevalent brain disease with high malignancy, mortality, and disability [1, 2]. Magnetic resonance imaging (MRI) is widely used in the clinical diagnosis of glioma, can clearly reflect the anatomical structure of human soft tissue, and can accurately display the location, size and histological characteristics of lesions. The segmentation of glioma lesions are the key steps for computer-aided diagnosis, surgery, radiotherapy, and chemotherapy planning of brain glioma.

Gliomas show infiltrative growth with lack of clear boundary and fixed growth pattern. Complex pathological changes, such as hemorrhage, necrosis, and edema, are found inside tumors. Gliomas show complex changes in brightness and texture on MRI images because of the complex pathological changes. Different tissues may have similar gray values, which present challenges to the accurate, repeatable, and stable segmentation of gliomas.

In clinical application, radiologists mainly perform manual segmentation, which is subjective, has heavy workload, and difficult to achieve repetitive segmentation. Some semi-automatic segmentation algorithms can segment ROI with minimal human interaction, integrate the advantages of manual and automatic segmentation, and improve segmentation efficiency. However, applying the scene that needs automated processing is difficult because of the necessity to set initial seed points, thresholds, and iteration termination conditions, among others. Automatic segmentation is completely controlled by an algorithm without human interaction. The segmentation speed is high and the results are repeatable. Automatic segmentation is conducive to the end-to-end application development of glioma [3]. Automatic segmentation is the main research direction of glioma segmentation, while improving the accuracy of segmentation is the key challenge.

In clinical practice, radiologists make a comprehensive diagnosis of glioma on the basis of the characteristics of various MRIs. Commonly used sequences generally include at least four types: T1-weighted imaging (T1), T2-weighted imaging (T2), fluid-attenuated inversion recovery (FLAIR) imaging, and contrast-enhanced T1-weighted (CET1) imaging [4, 5]. CET1 can reflect the blood flow information of a lesion, T1 provides anatomical information, FLAIR imaging can help distinguish the cerebrospinal fluid of the edema area, and T2 is sensitive to the edema area and can provide such information as tumor boundary and edema degree [6]. In these sequences, T2 images can considerably reflect the morphological information of tumors and are often used in clinical segmentation of gliomas. Moreover, using the segmentation result of the edema area as ROI of each sequence can provide information on all types of regions of tumors because such an area often contains the real and necrotic areas of tumors. Therefore, the design of an automatic segmentation algorithm for T2 sequence has superior clinical value.

## Related studies

Glioma segmentation algorithms can be divided into region-based, edge-constrained, classification or clustering, and some hybrid methods [7].

Region-based methods obtain the segmentation result by iteratively adding adjacent to the seed points through similarity evaluation. Region growth algorithms is a typical representation of the region-based methods, while the key points are the design of similar metrics and growth rules [8]. The disadvantage is that the seed points should be specified manually. Ref. [9] implements the initialization of algorithm by mapping a tumor with multi-spectral histogram. Ref. [10] proposes the semi-automatic segmentation algorithm LuTA for lung cancer tumor using region growth algorithm. Ref. [11] extends LuTA by developing an automatic seed point generation algorithm, which automatically generates multi-seed points in the core region of tumors to achieve automatic segmentation.

The edge-constrained methods use the feature that the target object’s gray level changes substantially in the edge position to segment ROI. An active contour model can maximize the related prior knowledge to constrain the image segmentation process. Snake model is one of the representative algorithms of the active contour model, which considers image segmentation as an energy minimization problem [12]. Brightness and texture features in Ref. [13] are used to drive the active contour to approach the tumor boundary in different MRI sequences. The level set segmentation algorithm implicitly represents the evolution curve as a zero-level set of high dimensional level set functions, which has a good theoretical basis, can be rapidly extended to three-dimensional segmentation and is extensively used in the segmentation of gliomas [14–16]. Chan and Vese construct external forces to guide the evolution of curves by mean values of the inner and outer regions, and proposed a level set method Chan-Vese (CV) based on the global regional gray mean value [17]. However, the problem of edge overflow is prevalent using the level set segmentation algorithm, which needs to be further deepened and improved, owing to the heterogeneity of glioma and unclear boundary between tissues.

The classification or clustering methods divide a group of objects into several categories on the basis of the simple and intuitive principle that intra- and inter-class distances are small and large, respectively. Ref. [18] explores the user of K-means clustering for segmentation of different brain tissues. Ref. [19] uses SVM in the segmentation of brain tumors. Deep neural networks are also used for brain tumor segmentation [20–22]. In Ref. [22], a customized convolutional neural network is used to segment glioblastoma, thereby achieving automation. However, deep learning remains difficult to apply in glioma segmentation because of the difficulty of obtaining high-quality glioma samples. Ref. [23] applies a random forest with context-aware features to identify sub-regions of tumors in MRI images. Given the characteristics of the random forest algorithm, this algorithm’s repeatability is not high, while difficulty is observed in achieving the continuous improvement of the existing model for the new sample. Ref. [24] extends the multiplicative inner composition optimization algorithm and applies it to the semi-automatic segmentation of glioma. Although good results were achieved, the interaction of the radiologist remains necessary. However, additional information may lead to other bias, which needs to be carefully screened in model selection.

Superpixel-based glioma segmentation is a hybrid method. This type of segmentation uses the clustering method to segment superpixels and trains classifiers on the basis of features calculated from each superpixel to classify tumor regions. Ref. [25] calculates the first-order intensity statistics features, Gabor textons, fractal analysis, and curvature features from each superpixel and realizes the detection and segmentation of brain tumors in the FLAIR images using the extremely randomized trees (ERT) classifier. It proves that the combination of superpixel segmentation and machine learning classification is feasible for brain tumor segmentation. Ref. [26] performed segmentation based on graphical models with a probability maximization framework, which uses conditional random field to model the spatial interactions among image superpixel regions. ℓ_1_-regularization was performed with statistics features, texture features, curve features and fractal features among objective function optimization. Ref. [27] proposed a superpixel segmentation to generate approximately structural superpixels with sharp boundary adherence and comprehensive semantic information, and applied the method to brain tissue segmentation to illustrate the superior performance. Ref. [28] presents an iterative spatial fuzzy clustering algorithm to generate 3D supervoxels for brain MRI volume based on prior knowledge of generating reliable seeds from a population-based brain template MRI image. Ref. [29] extracted supervoxels by the SLIC method and calculated intensity histogram, texture and shape features from each supervoxels, training a multinomial logistic regression by maximizing the mutual information between the data and their labels, and then automatically assigns a supervoxel with a class label.

For the segmentation based on superpixel, the distribution of superpixels will directly affect the segmentation results. Two parameters, namely, number of superpixels and compactness, are critical to the superpixel distribution. At present, hyper-parameters are mainly searched using grid or set default values, although obtaining the best parameter remains challenging. Ref. [25] set fixed parameters for SLIC algorithm with 1600 superpixels and compactness equals 0.2. Ref. [26] uses SLIC0 for superpixel segmentation, compactness is not needed, the search for the optimal number of superpixels is performed through grid search, the search range is from 40 to 260, step size is 20, compactness is set to 40, and the number of iterations set to 10. Since the new sample does not have ground truth, it is impossible to determine the optimal number of superpixels, so it can’t be used directly.

Superpixel segmentation preprocesses a pixel-level image into a block image by merging the homogeneous regions, thereby obtaining a few regions and effective spatial location information. This type of segmentation can effectively reduce the difficulty of post-processing and improve the robustness of the algorithm. After the glioma image is segmented by superpixel, ROI is concentrated in at least one adjacent superpixel block. The heterogeneity of brightness and texture on the superpixel block reflects the heterogeneity of tumors. The statistical, texture, curvature, and fractal features of each superpixel block are well distinguished. We can realize the segmentation of ROI by classifying the superpixel blocks using machine learning method. This study analyzes the automatic segmentation method of glioma using superpixels. The main contributions of this research are as follows.

1. Given that the simple linear iterative clustering version with 0 parameter (SLIC0) algorithm needs to set the hyper-parameter of the number of superpixels, this study proposes the adaptive SLIC0 (ASLIC0) algorithm, which can automatically estimate the optimal number of superpixels. The proposed algorithm achieves a fully automatic and efficient superpixel segmentation in the T2 images. Moreover, ASLIC0 does not need human intervention and has good stability and repeatability.
2. The current research aims to design a framework for the segmentation of glioma using superpixel features and machine learning. We compute the statistical, texture, curvature, and fractal features for each superpixel. Thereafter, the classifier is trained using SVM.

## Methods

**Fig. 1:**
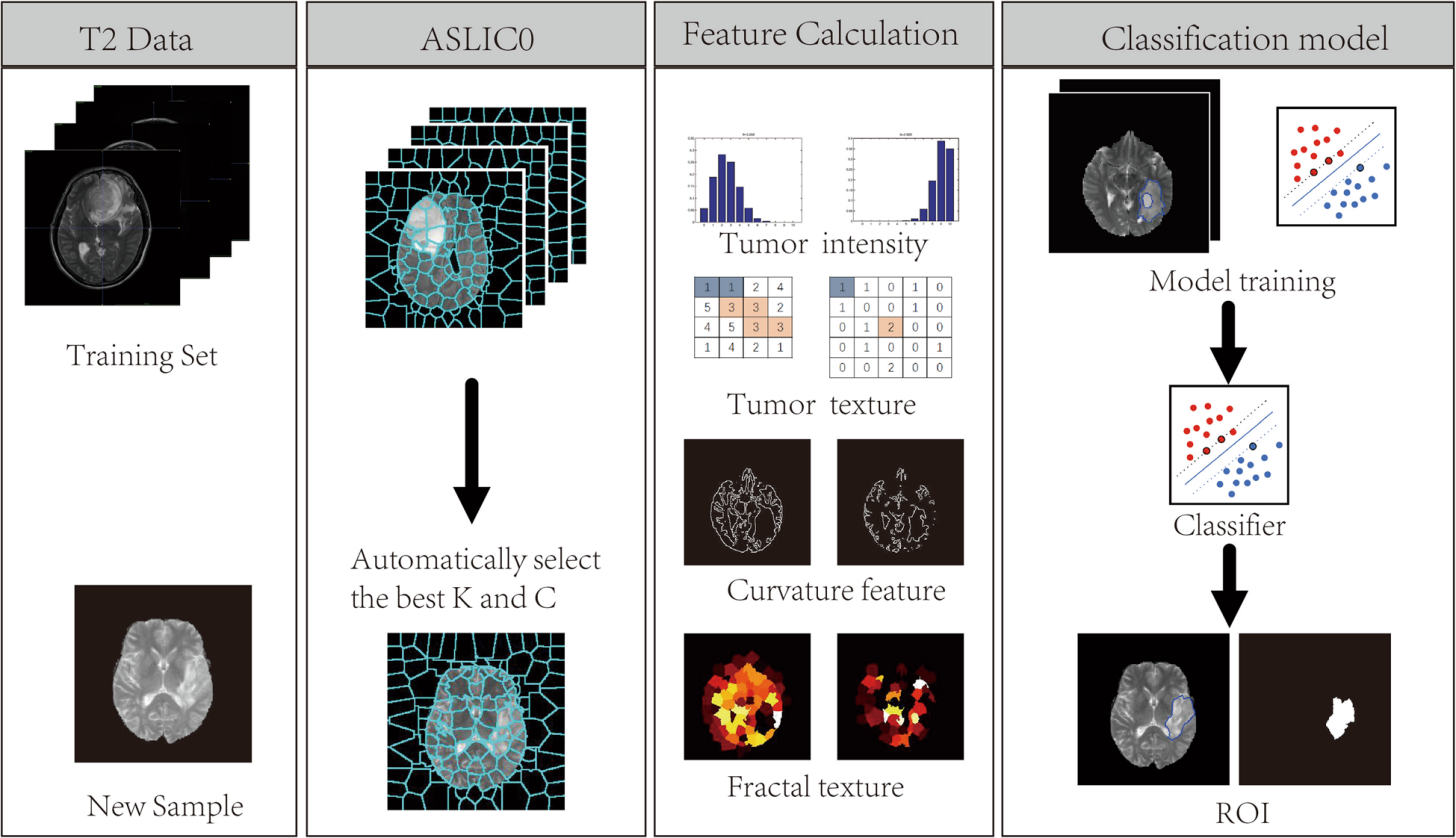
Flowchart of glioma segmentation based on superpixel

Automatic glioma segmentation based on adaptive superpixel adopts the following steps: (1) Perform ASLIC0 to obtain superpixel images that fit well with the tumor boundary and have only a few superpixels; (2) Generate the training set by calculating the features and label for each superpixel; and (3) Train the classifier using the SVM model and classify the superpixel into tumor or non-tumor regions. Figure 1 shows the flowchart of the proposed framework.

### Adaptive superpixel partitioning

The simple linear iterative clustering (SLIC) [30] method partitions images into superpixel patches using the k-means clustering algorithm. The distance measure in clustering algorithm includes the distance of the gray space *d*_*c*_ and Euclidean distance of pixels *d*_*s*_. The calculation formulas are (Eqs. 1 and 2), respectively. The distance D of two pixels is calculated using Eq. (3).

1 $$ {d}_c=\sqrt{{\left({I}_i-{I}_j\right)}^2} $$

where *I*_*i*_ and *I*_*j*_ are the gray values of pixels i and j, respectively.

2 $$ {d}_s=\sqrt{{\left({x}_i-{x}_j\right)}^2+{\left({y}_i-{y}_j\right)}^2} $$

where x and y are the coordinate values.

3 $$ D=\sqrt{{d_c}^2+{\left(\raisebox{1ex}{${d}_s$}\!\left/ \!\raisebox{-1ex}{$S$}\right.\right)}^2{C}^2} $$

In Eq. (3), parameter S is used to limit the range of local clustering. In the SLIC algorithm, $S=\sqrt{N/K}$, where N is the total number of images and K is the number of superpixels. Compactness C can be used as a balance parameter to adjust the relationship between color distance and spatial distance. Equation (3) shows that the parameters that should be set manually in SLIC include K and C. Accordingly, the selection of these two parameters will directly affect the performance of segmentation. Parameter K determines the number range of irregular regions; the larger K is, the smaller the size of the superpixel block is. Compactness C determines the proportion of spatial distance. When C is large, the proportion of spatial distance will increase, while the superpixel will have a smooth boundary. When C is small, the boundary of superpixel will be close to the edge of the image but the shape and size will be considerably irregular. Therefore, the key to the effective implementation of the SLIC algorithm is to find the best K and C.

**Fig. 2:**
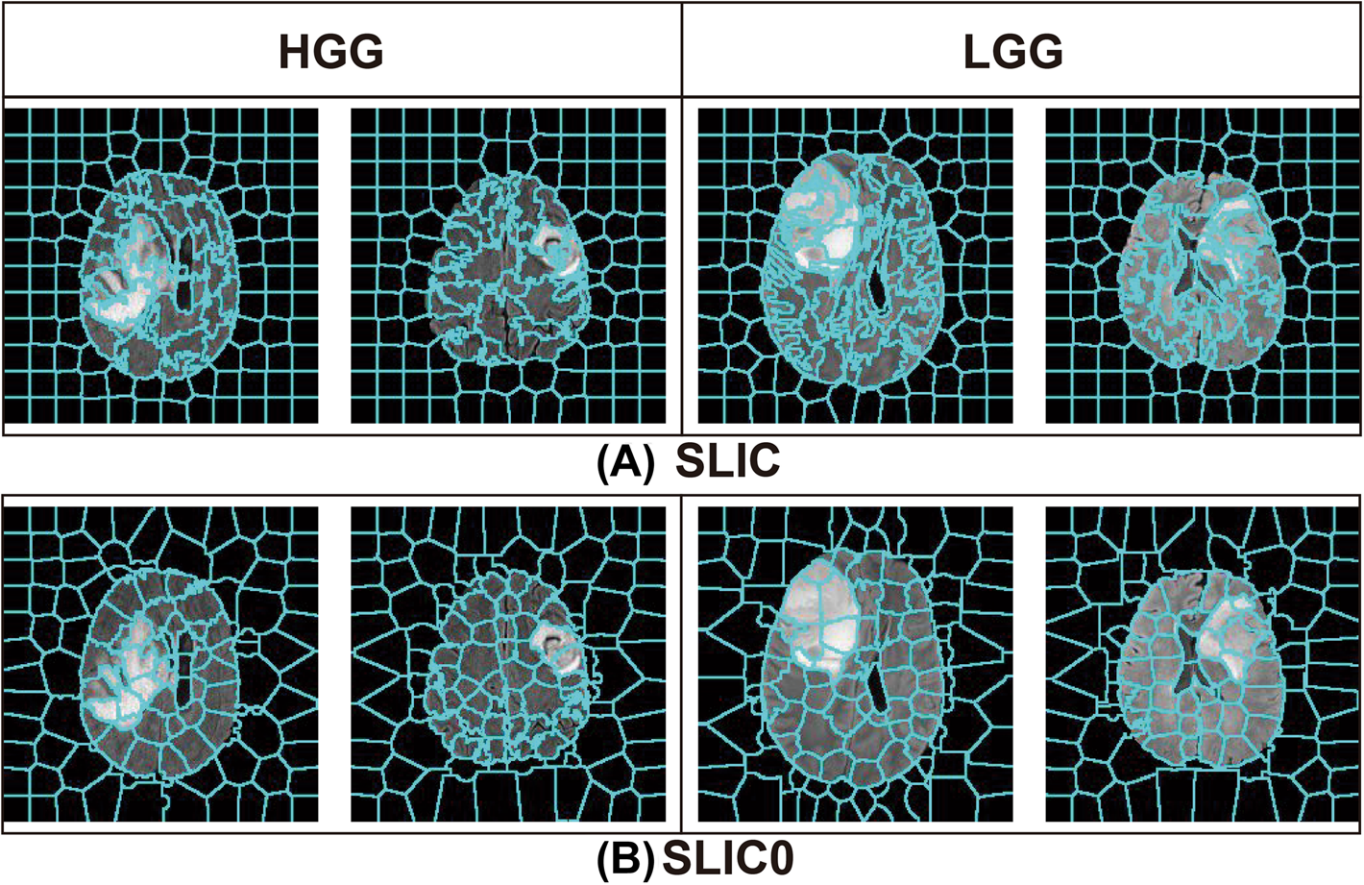
Comparison of SLIC and SLIC0

The SLIC with 0 parameter (SLIC0) algorithm [31, 32] improves the selection of the C value by changing the fixed value of SLIC to an adaptive value for each superpixel in the first iteration, thereby solving the problem of the C parameter selection. Experiments on the T2-weighted MR images of gliomas show that the SLIC0 algorithm can generate relatively regular superpixels in flat and highly variable regions and bring the superpixel close to the tumors or edema regions. Figure 2 shows that for high-grade gliomas (HGG) and low-grade gliomas (LGG), the superpixel blocks obtained by the SLIC0 algorithm can substantially fit the tumor boundary. Therefore, the SLIC0 algorithm solves the problem of hyper-parameter C selection, thereby resulting in the key problem of superpixel segmentation becoming the problem of the hyper-parameter K selection. This study proposes an ASLIC0 algorithm, while the automatic selection of the hyper-parameter K is realized.

**Fig. 3:**
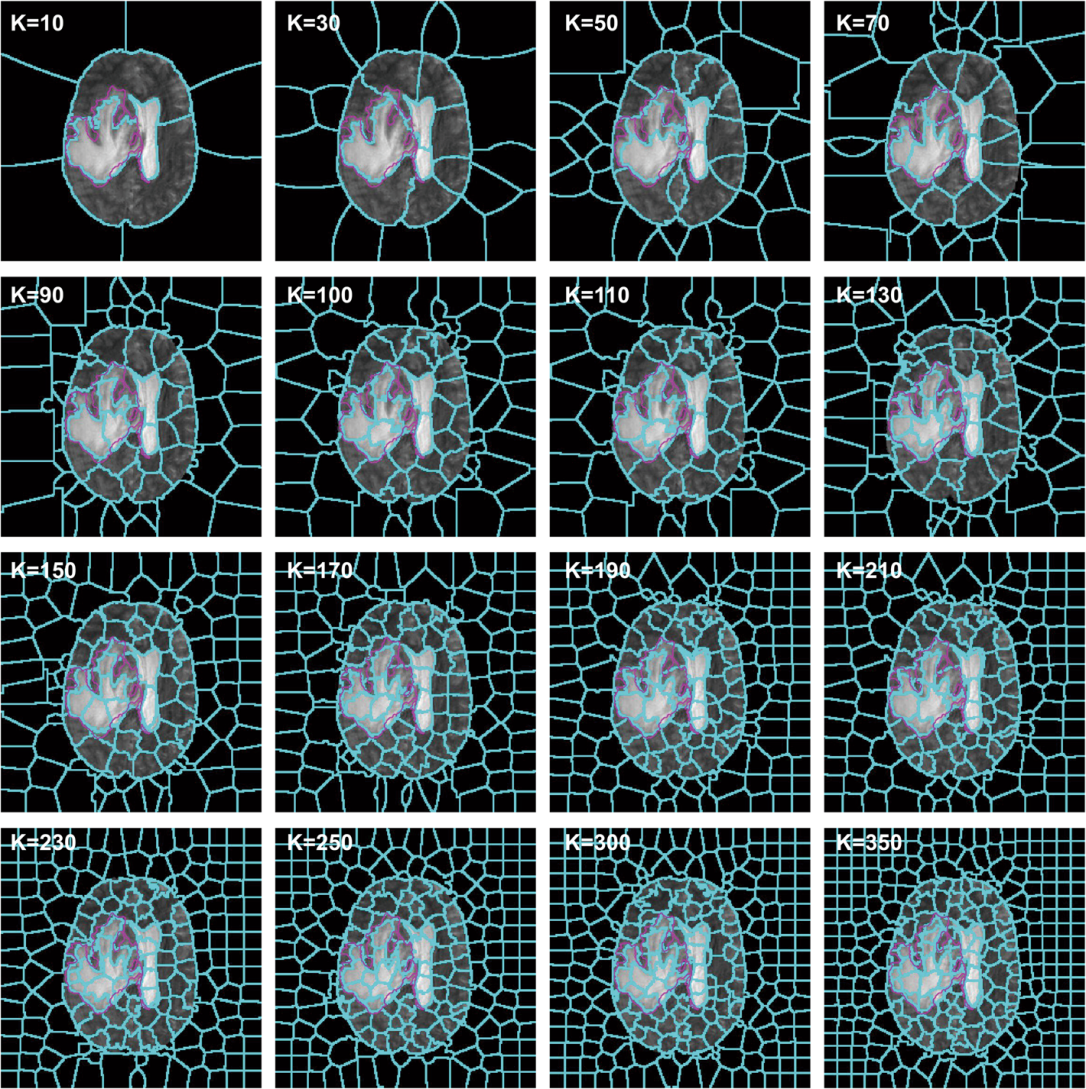
Effects of different K on superpixels

Experiments show that the hyper-parameter K is directly related to the segmentation effect. When K is considerably small, the size of the superpixel blocks is substantially large, thereby possibly leading to difficulty in distinguishing ROI. If K is considerably large, then the size of each superpixel will be substantially small, thereby resulting in each superpixel block losing regional features. This loss results in the lack of representativeness of the features that will be eventually calculated, thereby leading to the reduction of segmentation accuracy. Given the increase in superpixel blocks, the demand for computing resources will increase, while the advantages of the superpixel segmentation will be reduced. Figure 3 shows the segmentation effect under a various number of superpixels. The blue line is the superpixel boundary, while the pink line circles the ROI boundary. Evidently, the increase in the number of superpixels results in the boundary between superpixels and ROI becoming increasingly consistent with the actual situation. When the number of superpixels exceeds 150, the change of superpixels in the ROI is not evident.

Numerous observations have shown that the best value of the hyper-parametric K is directly related to the size of the tumor and complexity of the tumor boundary. To find the best value of the hyper-parameter K, which can segment the ROI well while the value is small, this study designed two evaluation indices, namely, area ratio of tumors (TAR) and boundary complexity of tumors (TC). Moreover, TAR and TC are combined with the best K value labeled by hand to form a training set. The K value of the new sample is predicted by training the random forest model.

#### Evaluation index of tumor

**Fig. 4:**
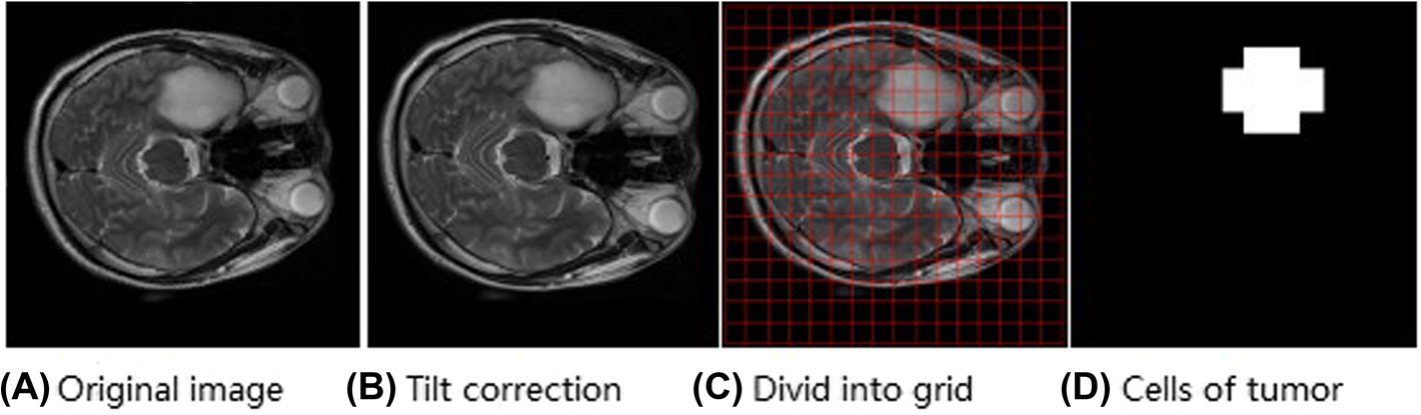
Finding of suspected tumor areas

This research used TAR to reflect the volume of tumors. However, the method of calibrating the region of the tumor becomes a key problem that should be solved because the image does not know the location information of the tumor before segmentation. The normal brain tissue is a symmetrical structure because of the particularity of the head, while the appearance of a tumor tissue breaks this symmetry. Therefore, we look for the asymmetrical region in the left and right hemispheres as the tumor area. Figure 4 shows the method of finding suspected tumor areas.

The following steps are performed on the original image (Fig. 4 (A)).

1. Image correction is performed, in which the majority of the areas of the corrected image are symmetrical with respect to the midline (Fig. 4 (B)) [33].
2. The entire image is divided into an average of 256 grids by placing a grid on the image (Fig. 4 (C)). The horizontal and vertical grids are divided into 16 equal parts (15 pixels each).
3. Equation 4 is used to calculate the similarity of the pixels in the corresponding grids of the left hemispheres (A) and right hemispheres (B) of the brain.

4 $$ r=\frac{\sum \limits_m\sum \limits_n\left({A}_{mn}-\overline{A}\right)\left({B}_{mn}-\overline{B}\right)}{\sqrt{\sum \limits_m\sum \limits_n{\left({A}_{mn}-\overline{A}\right)}^2{\left({B}_{mn}-\overline{B}\right)}^2}} $$

Where m and n represent the size of the grid, all of them were 15 in this study.

(4) When the similarity r is below the threshold (threshold is 0.81), both regions in the left and right hemispheres are considered suspected tumor areas (Fig. 4 (C)). Lastly, the number of blocks in left hemispheres is counted as n.
(5) TAR is calculated by *T*A*R* = *n*/256.
(6) The tumor boundary complexity index TC can be measured using the ratio of the perimeter to the area of the tumor. For the suspected tumor area calculated in step 4, TC is calculated using Eq. 5. To calculate conveniently, the number of pixels in the boundary is used to replace the perimeter, while the number of pixels in the suspected area is used to replace the area.

5 $$ TC= Perimeter/ Area\approx N(edge)/N(area) $$

The function *N* is used to calculate the number of pixels.

#### Evaluation of the best value of K

For each image in BraTS2017, the optimal hyper-parameter K is searched through a grid search, in which the search range is from 10 to 450, while the search step is 10.

For the superpixel image of each K in the grid search, the proportion of each superpixel to the manual segmentation (ground truth) is calculated. The superpixels with a ratio of over 90% are merged into the superpixel ROI, which is denoted as variable A. The result of the ground truth is denoted as variable B. The Dice index is calculated using Eq. 6 [34].

6 $$ Dice\left(A,B\right)=\frac{2S\left(A\cap B\right)}{S(A)+S(B)} $$

Function *S* denotes the area. The closer the value of Dice is to 1, the better the effect of K is.

**Fig. 5:**
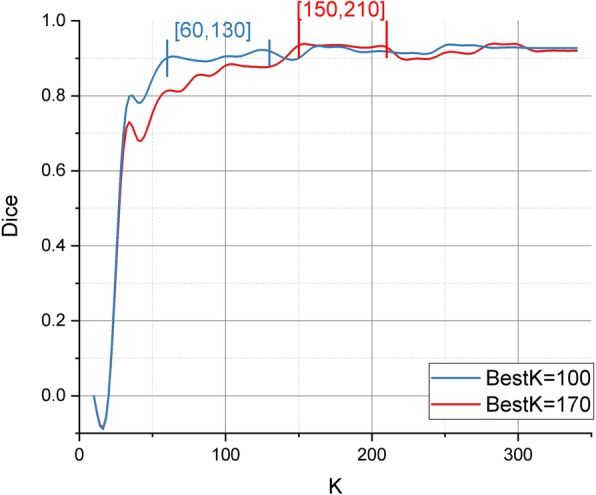
Effect of the number of superpixel K on the Dice index

The Dice index indicates that the performance of the different K in the grid search is evaluated. For the same image, the experimental results show that good results can be obtained in a certain range of K. Figure 5 shows that for image 1 (blue), the result of Dice is approximately the same when K is above 60 but below 130. For image 2 (red), when K is above 150 but below 210, the result of Dice is approximately the same. This observation indicates that to make K have better robustness, the manual selection of K in the middle is a good choice. For the two images in Fig. 5, 100 and 170 are selected.

#### Prediction model of K

**Fig. 6:**
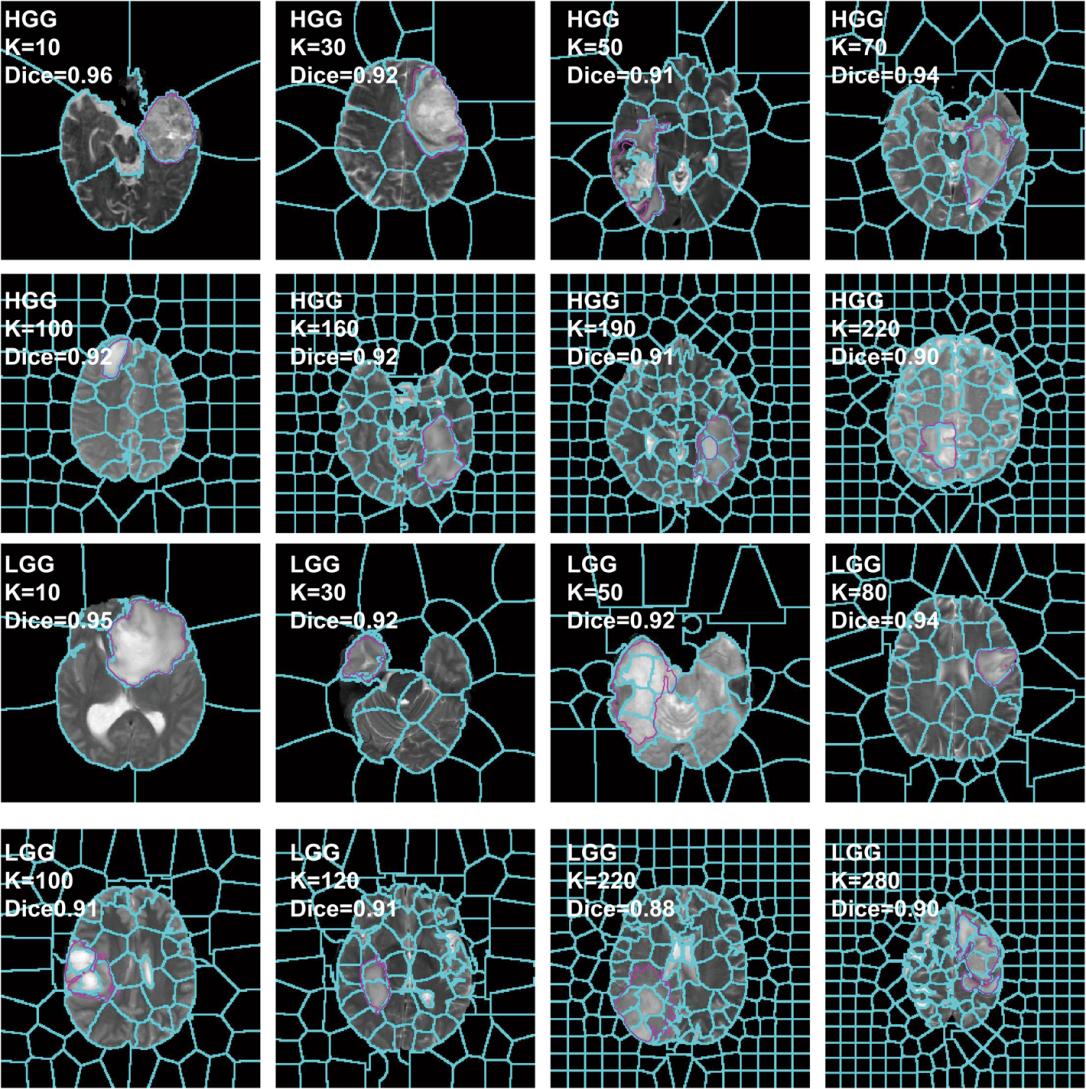
Selection of K for the different glioma images

The best choice of K is extremely different for the different glioma images. Figure 6 lists several examples of high-grade glioma (HGG) and low-grade glioma (LGG) and shows no fixed pattern for the selection of K. Thus, we need to determine the applicable rules through machine learning. For all BraTS2017 images containing tumors, TAR and TC are calculated, while K is manually selected as the target value. Thereafter, a regression model is trained using random forest. We will use the trained model to predict K.

### Superpixel annotation and feature extraction

#### Image preprocessing

Numerous black backgrounds apart from the head are observed in brain MRI images. Background does not make sense for research but increases computation. Therefore, the background should be removed before the feature calculation to reduce the impact of the background.

Overall, the brightness of the background is small, although there may be some large values because of the noise. The influence of noise can be eliminated by calculating the mean value of the superpixel. This study uses the threshold method to remove background. When the average brightness of pixels in the superpixel is below the threshold value, the superpixel will be removed. The threshold used in this study is that the average brightness is below 5.

#### Superpixel annotation

**Fig. 7:**
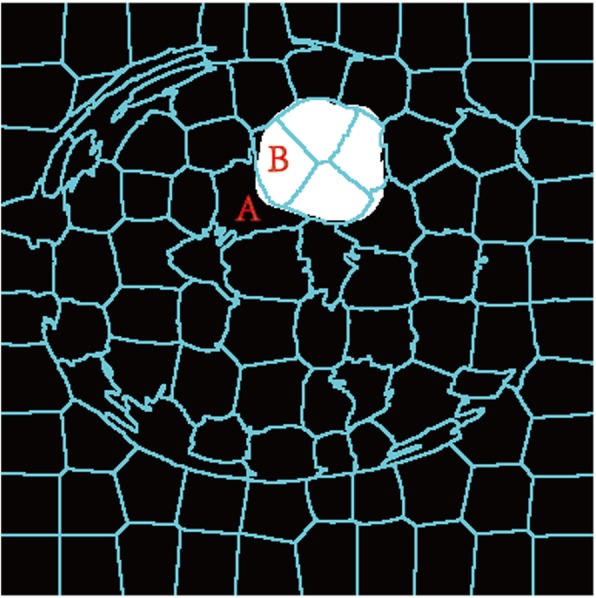
Superpixel auto-annotation

To train the superpixel classification model, whether the superpixel is a tumor area should be known to determine the performance of the classifier. This research uses manual segmentation as ground truth, while the Dice similarity coefficient is used to determine whether a superpixel belongs to a tumor area. When the Dice value is above the threshold value, it is determined as the tumor area; otherwise, it is a non-tumor area. For example, superpixel A in Fig. 7 does not belong to the tumor region because of the small proportion of this tumor region. By contrast, superpixel B belongs to the tumor region because the proportion of this region is above the threshold. Threshold vary slightly depending on the image sequence.

#### Feature extraction

Feature extraction aims to quantify each superpixel. The intensity distribution of the pixels inside the superpixel should be transformed into comparable features. The features in this study include intensity statistical, texture, curvature, and fractal features, totaling 69.

##### Intensity statistical features

Intensity statistical features are based on the first-order statistics of histogram and describes the distribution of values without considering their spatial relationship, including 17 statistical features (e.g., energy, entropy, mean, maximum, and median). Standard deviation, variation, and mean absolute deviation are used to evaluate the dispersion degree of statistical histogram. Variation, kurtosis, and skewness are commonly used central moments. In particular, skewness reflects the asymmetry degree of the statistical histogram deviating from the mean, while kurtosis reflects the steepness and slowness of the histogram distribution. Uniformity and entropy are calculated to measure the randomness degree of the histogram.

##### Texture features

The texture is an important visual cue, which is ubiquitous and difficult to describe in images. Gray level co-occurrence matrix (GLCM) describes the second-order joint probability function of an image. A variety of GLCMs can be obtained from different directions and asynchronous lengths, while the corresponding Haralick texture features can be calculated [35]. Ref. [36] discusses that some texture information will be lost in the original images because of the influence of noise. Multilevel partial derivative mapping images can obtain images with rich texture structure [36, 37]. This study calculates GLCM from the original image, the gradient mapping map is calculated using the Roberts operator, and the gray level gradient co-occurrence matrix (GLGCM) is calculated, the curvature mapping map is calculated using the Prewitt operator, and the gray level curvature co-occurrence matrix (GLCCM) is calculated. The Haralick texture features are calculated for the GLCM, GLGCM, and GLCCM matrices.

##### Curvature feature

Curvature feature is a shape-based feature in image, and a measure of image irregularity. This feature is widely used in computer vision.

For image *I*, if *f*_*x*_ and *f*_*y*_ represent the gradient of the image along with the x and y directions respectively, *f*_*xx*_ and *f*_*yy*_ are the second-order partial derivatives of image *I*(*x*, *y*). Accordingly, the curvature of the image is calculated using Eq. 7.

7 $$ Curv=\frac{f_{xx}{f}_y^2+{f}_{yy}{f}_x^2-2{f}_{xx}{f}_x{f}_y}{{\left({f}_x^2+{f}_y^2\right)}^{\raisebox{1ex}{$3$}\!\left/ \!\raisebox{-1ex}{$2$}\right.}} $$

Given that the curvature is calculated for a single pixel, the curvature feature in a superpixel is defined as the average curvature of all the pixels in the superpixel.

##### Fractal features

**Fig. 8:**
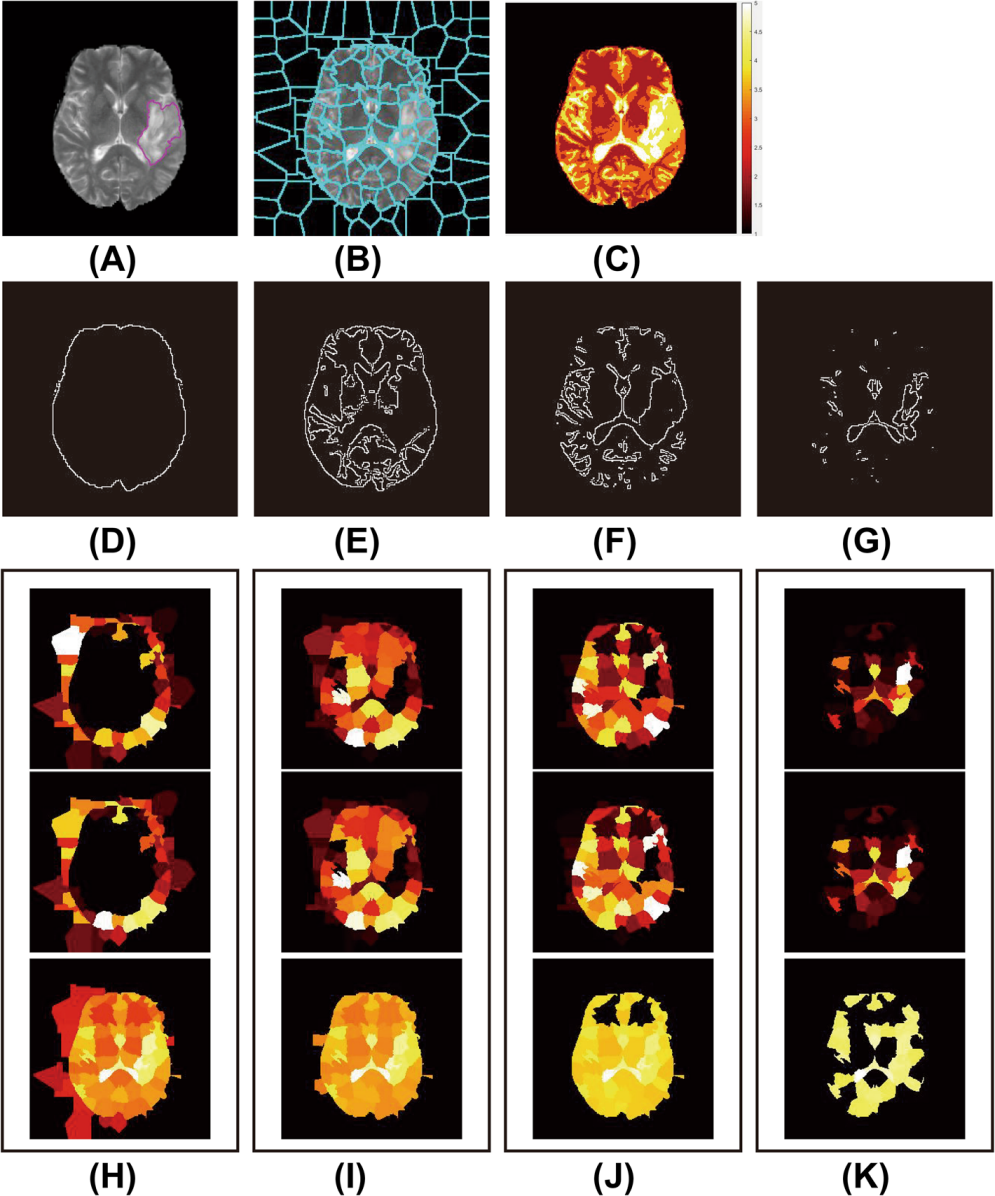
Process of extracting fractal features

Given the heterogeneity of gliomas, the tumor area shows complex structures and irregularities, while the normal brain tissue is regular and smooth. Moreover, the tumor area also shows similar structure and roughness. By utilizing the characteristics of the fractal features, the corresponding superpixels at a certain scale in the tumor area have self-similarity and good distinction between the tumor lesion and normal tissue. The current study uses the Otsu algorithm [38] to obtain binary images with different thresholds, while the fractal edge information of each binary image is obtained. we choose 4 as the number of channels to obtain good results and reduce the computational complexity in the implementation of the Otsu algorithm. Thereafter, the fractal features corresponding to superpixels are calculated (D, E, F and G in Fig. 8). The fractal features include the area, average brightness (rows 1, 2 and 3 of columns G, I, J and K in Fig. 8), and fractal distance, which is obtained using the box algorithm [39]. The process of extracting fractal features is shown in Fig. 8.

### Machine learning tumor segmentation

For the feature set calculated from each superpixel, the machine learning algorithm is used to train a classification model to classify the tumors and non-tumors, thereby achieving the segmentation of glioma. As a classical classification algorithm, SVM is widely used in machine learning, computer vision, and data mining. The basic idea of SVM is to find the maximum separating hyperplane in the sample space of the training set and separate the training samples. This research uses SVM as a classifier because the final model is only related to the support vector and over-fitting is difficult to produce.

This study uses the statistical and machine learning toolkit of MATLAB to train the SVM classifier. Bayesian optimization is used in the training process, while radial basis function (RBF) is used as kernel function. The training data are standardized among the training processing. In all data, 80% of the samples were used as training set (228 cases) and 20% samples were retained as test set (57 cases). During the model training, 5-folds cross-validation is used to select the final model.

## Results

### Data set

For convenience, this study uses the public data set BraTS2017 [6, 40], the data sources of which include the Cancer Genome Atlas and Cancer Imaging Archive. All data include the T1, CET1, T2, and FLAIR sequences and have been pre-processed with the same resolution and registered with the same anatomical template. This research is based on the 2D image of the T2 sequence. BraTS2017 contains 285 preoperative glioma samples, include 210 HGG samples and 75 LGG samples. Although each sample contains 155 slices, these images are obtained by head registration with fewer original slices. To ensure the representativeness of the samples, the slice of the median of the tumor pixels in each sample is used as the training image.

### Selection of parameters

To predict the value of K, a regression model is trained by random forest using TAR and TC, while K is manually selected as a training set. R-square is used to evaluate the fit between the predicted K value and manually selected K value. R-square is calculated using Eq. 8.

8 $$ {R}^2=1-\frac{\sum {\left({k}_{best}-{k}_{predict}\right)}^2}{\sum \left({k}_{best}-\overline{k_{best}}\right)} $$

The denominator represents the dispersion of K, while the numerator represents the error between the predicted result and best K. R-square can substantially evaluate the regression model. Accordingly, the closer its value to 1, the better result will be obtained.

In the random forest training, the number of trees is 5, while the maximum tree depth is not set. When the maximum number of instances exceeds 4, the nodes are stopped from splitting.

**Fig. 9:**
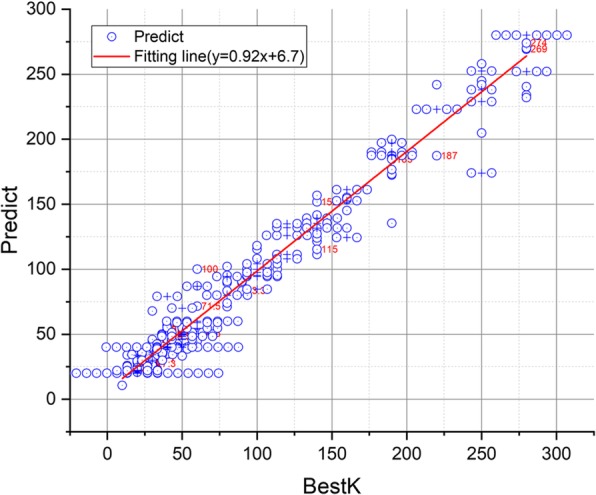
Scatter plot of the best K and predicted values

The experimental results show that the final training model can fit K substantially, while R-square is 0.9719. Figure 9 shows the scatter plot of the best K and predicted values, while the red line is the fitting line. The closer the red line is to y = x, the better result will be obtained. The fitting curve of this experiment is y = 0.92x + 6.7.

Statistical features are directly calculated from the intensity of the pixel in each superpixel. Given that the original image is stored by 12 bits, the texture feature with such large gray value cannot considerably express the texture characteristics. Before calculating the texture features, gray resampling of the original brightness is necessary. This study uses 8, 16, 32, and 64 Gy levels to calculate the texture features. The calculation of GLCM is performed in the 0^°^, 45^°^, 90^°^, and 135^°^ directions, while the step is 1. Curvature feature is calculated from the intensity of the pixel, while the mean value is used as the feature of the corresponding superpixel. Fractal features are calculated using four channels.

### Segmentation results

During the test process, the test samples are processed into superpixel images by ASLIC0 algorithm, and four kinds of features are calculated for each superpixel. Superpixels predicted as tumor regions constitute the final segmentation. For all segmentation result of the test set, the average value of Dice, and Hausdorff distance (HD) [28], sensitivity and specificity were calculated to evaluate the algorithm.

Table 1 shows the segmentation performance of our method on BraTS2017. The method presented in this study has a good performance for images with high contrast between tumor areas and normal tissues in glioma images. Moreover, good results can be obtained on the HGG and LGG samples.

**Table 1:** Segmentation performance of the proposed method on BraTS2017

Samples	Dice	HD (pixels)	Sensitivity	Specificity
LGG	0.8566 ± 0.07	3.4419 ± 0.75	82.58 ± 10.45%	99.61 ± 0.42%
HGG	0.8463 ± 0.08	3.4805 ± 0.68	81.04 ± 10.83%	99.64 ± 0.36%
ALL	0.8492 ± 0.07	3.4697 ± 0.69	81.47 ± 10.75%	99.64 ± 0.38%

**Fig. 10:**
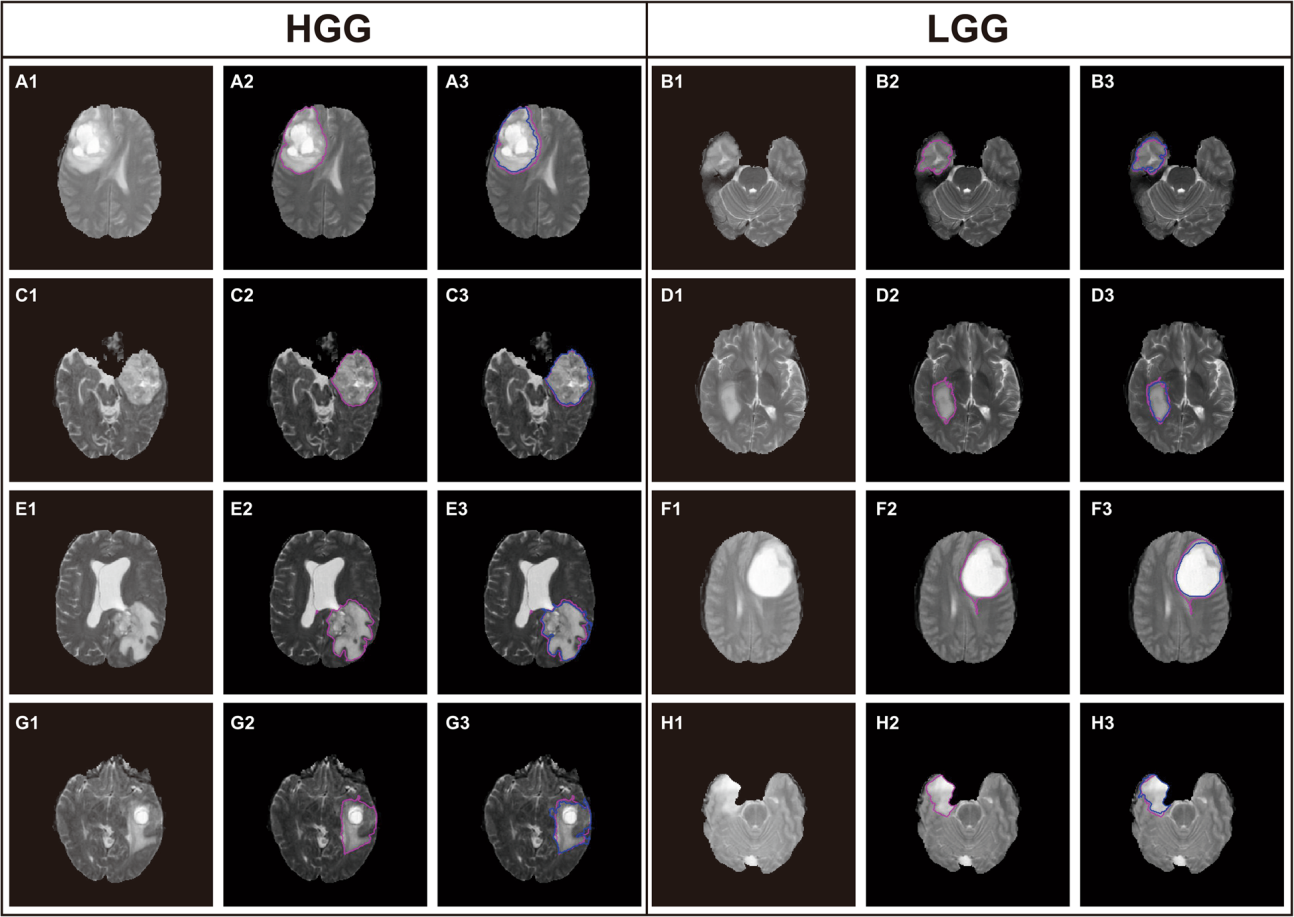
Segmentation results of the proposed method

Figure 10 shows some segmentation results. Each row from top to bottom represents samples with different positions, brightness contrast, and tumor appearance. The left three columns are HGG samples, while the right three columns are LGG samples. Sublabels 1 and 2 in the three columns represent the original image and boundary of the ground truth, respectively. Sublabel 3 shows the comparison between the boundary of the segmentation result and ground truth. The blue and red lines represent the segmentation result and ground truth, respectively. Overall, the majority of the segmentation results have good performance.

Observations also show that the segmentation boundaries are slightly closer to the inner pixels than the ground truth. This result is related to the tendency of manual segmentation and characteristics of superpixel segmentation. In the T2 sequence, the brightness of the tumor area is often high, while the color of brain tissue around the tumor is relatively dark. In manual segmentation, the edge is often placed in the low-brightness region to ensure that the entire tumor area can be included. In superpixel clustering, the brightness distance is dominant at the edge position. Thus, the edge will be placed in the high-brightness region, thereby enabling the segmentation results to be closer to the actual tumor area than the manual segmentation results. This outcome will not affect the actual clinical application. If necessary, the boundary can be expanded by 1 to 3 pixels through post-processing.

### Comparative experimental results

To compare the performance of the methods, the authors use classical segmentation algorithms and similar algorithms for comparative experiments. These algorithms include classical segmentation Snake [12], region growth [11], the classical level set method Chan–Vese (CV) [17], and two superpixel-based algorithms [25, 26]. All experiments use the same origin data. The seed of the region growth is initiated at the mean coordinates of ground truth, while the initiate level set function around the same seed by a circle with a diameter of 2 pixels and initiate the snake mask around the seed by 2 pixels. For the experiments in Ref. [25], K and compactness are set to 1600 and 0.2, respectively. For the experiments in Ref. [26], the search for K is performed through grid search, the search range is from 40 to 260, step size is 20, compactness is set to 40, and the number of iterations set to 10. Table 2 shows the comparison results.

**Table 2:** Comparison with other related methods using BraTS2017

References	Methods description	Dice	HD (pixels)
Proposed Methods	Automatic SLIC0 + SVM	0.8492	3.47
Snake [12]	Classical algorithm	0.6951	5.75
RegionGrow [11]	Classical algorithm	0.3764	8.37
CV [17]	Classical algorithm	0.4247	7.14
Soltaninejad et al. [25]	ERT + SLIC	0.8263	3.62
Zhe Zhao et al. [26]	SLIC + CRF	0.8052	3.83

The experimental results show that the proposed method is superior to traditional methods, such as snake, region growth, and CV. Competence in automated scenarios is difficult to achieve because of the heterogeneity of glioma and the need for the hyper-parameter selection of the classical algorithms, its iterative evolution mechanism is more suitable for better use in semi-automated scenarios.

The performance of the proposed method is slightly higher than the methods of Refs. [25, 26]. The reason may be that the prediction algorithm of the optimal hyper-parameter K improves the quality of the superpixels. Ref. [25] chooses the number of hyper-parameters by grid search, in which the search step is 20. However, the best hyper-parameter K may not be found because the search step is considerably large. Using fixed K and C in Ref. [26] is feasible, although achieving optimal results is difficult. Evidently, Fig. 5 shows that when the number of K exceeds a certain value, the performance decreases slightly but essentially remains unchanged.

## Discussions

**Fig. 11:**
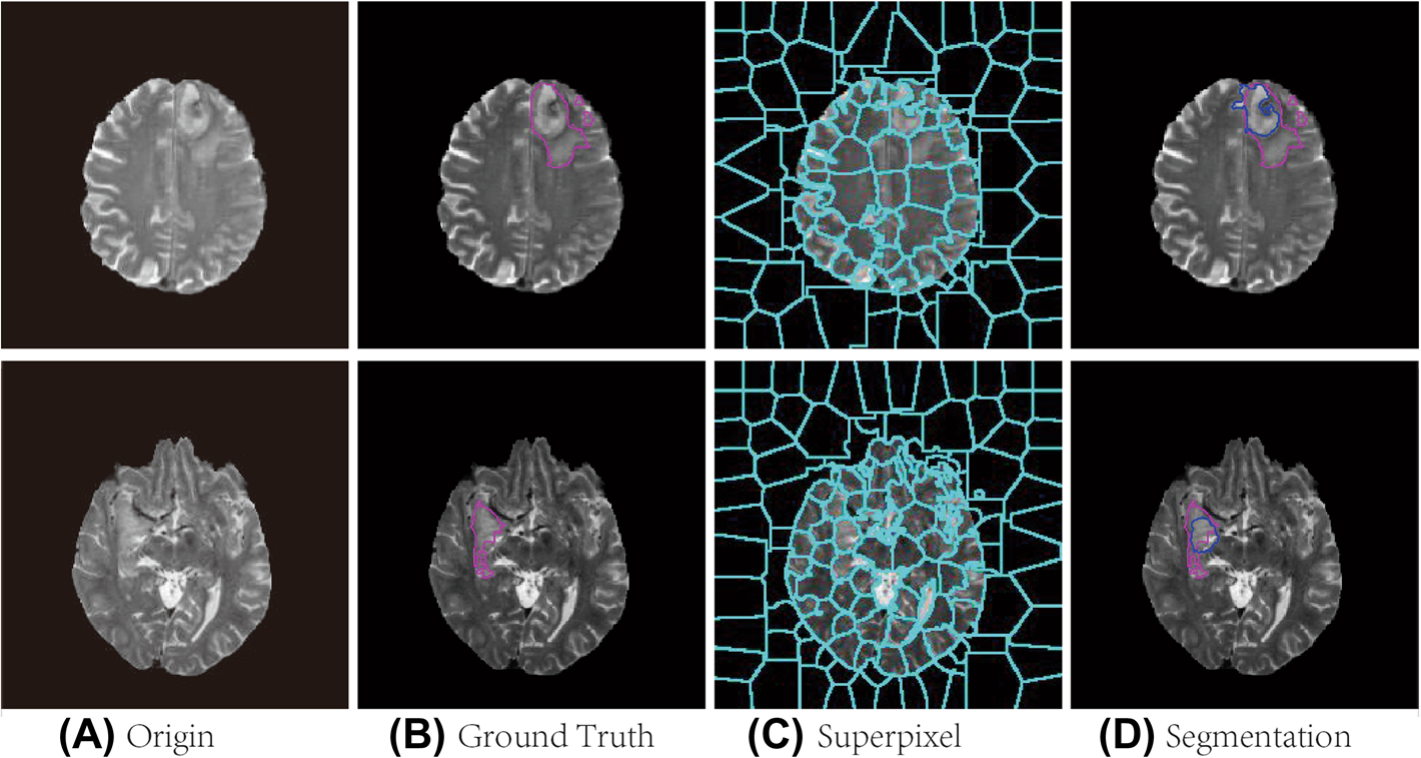
Examples of method ineffectiveness

The proposed method works well for most of the time, but the effect become worse when image comes to have blurred edges, or tumor area presents a large number of the cross with other normal tissues, or ROI’s gray level contains much overlapping with neighboring tissues. Figure 11 shows two examples of method ineffectiveness. In the first row, the contrast between the core area and the edema area is large, and the contrast between the edema area and normal brain tissue is not obvious. Therefore, the edema area does not generate superpixel, which makes the final classification model unable to work well, and Dice equals 0.5505. Compare to ground truth, our method succeeded in finding the core area. In the second row, tumor areas have no distinct boundaries and are poorly differentiated from surrounding tissues, similar to the first row, our method also cannot produce good segmentation, the Dice equals 0.4938. In these cases, improving the accuracy of superpixel partitioning remains the main challenge.

Brats2017 contains four sequences: T1, T2, FLAIR and CET1. Experiments show that our method works well on T2, FLAIR. We made a preliminary analysis, which may be due to the fact that on T2 and FLAIR, tumors are usually highlighted and their borders are relatively clear, while on T1, the area of the tumor is dark and have much overlap with brain tissue. The segmentation of the T2 sequence in our method is actually the segmentation of edema area. The original MR image of glioma is 3D image, the adjacent information between slices may promoting the effect of segmentation and reduce the overall segmentation time. This research is based on the 2D image of the T2 sequence, but this method can be easily extended to 3D images. Our next research will focus on 3D images.

## Conclusions

This study proposes an automatic segmentation method for brain glioma on the basis of the T2 sequence of BraTS2017. For the segmentation of superpixel, an ASLIC0 algorithm based on SLIC0 is proposed. In the ASLIC0 algorithm, the optimal hyper-parameter K is predicted by training a random forest through the design of two evaluation indicators, namely, ratio of tumor area and complexity of tumor boundary. The experimental results show that the random forest model can considerably fit K, while R-square is 0.9719. Superpixel images with only a few superpixels and close to the tumor boundary can be obtained using ASLIC0. The SVM prediction model is trained by calculating the statistical, texture, curvature, and fractal features of each superpixel. Meanwhile, the automatic segmentation of glioma are realized by classifying superpixels into tumor or non-tumor types. The experimental results on BraTS2017 show that the proposed method has good segmentation performance. The average Dice was 0.8492, Hausdorff distance was 3.4697 pixels, and sensitivity and specificity were 81.47 and 99.64%, respectively. The proposed method has good performance on the HGG and LGG samples, thereby showing that the proposed algorithm has good stability. Comparative experimental results also show that the proposed algorithm has superior performance.

## Data Availability

All data generated or analyzed during this study are included in the Multimodal Brain Tumor Image Segmentation Benchmark 2017 (BraTS2017). You can get data at URL: https://www.med.upenn.edu/sbia/brats2017/data.html

## Abbreviations

ASLIC0: Adaptive superpixel generation algorithm based on simple linear iterative clustering version with 0 parameter
BraTS2017: Multimodal Brain Tumor Image Segmentation Benchmark 2017
CET1: Contrast-enhanced T1-weighted (CET1) imaging
ERT: Extremely randomized trees
FLAIR: Fluid-attenuated inversion recovery imaging
GLCCM: Gray level curvature co-occurrence matrix
GLCM: Gray level co-occurrence matrix
GLGCM: Gray level gradient co-occurrence matrix
HGG: High-grade glioma
LGG: Low-grade glioma
MRI: Magnetic resonance imaging
ROI: Region of interest
SVM: Support Vector Machine
T1: T1-weighted imaging
T2: T2-weighted imaging
TAR: Area ratio of tumors
TC: Boundary complexity of tumors (TC)
